# Methane Output of Tortoises: Its Contribution to Energy Loss Related to Herbivore Body Mass

**DOI:** 10.1371/journal.pone.0017628

**Published:** 2011-03-09

**Authors:** Ragna Franz, Carla R. Soliva, Michael Kreuzer, Jean-Michel Hatt, Samuel Furrer, Jürgen Hummel, Marcus Clauss

**Affiliations:** 1 Clinic for Zoo Animals, Exotic Pets and Wildlife, Vetsuisse Faculty, University of Zurich, Zurich, Switzerland; 2 Institute of Plant, Animal and Agroecosystem Sciences, Swiss Federal Institute of Technology, Zurich, Switzerland; 3 Zurich Zoo, Zurich, Switzerland; 4 Institute of Animal Science, Rheinische Freidrich-Wilhelms-Universität, Bonn, Germany; University of Western Ontario, Canada

## Abstract

An increase in body mass (M) is traditionally considered advantageous for herbivores in terms of digestive efficiency. However, recently increasing methane losses with increasing M were described in mammals. To test this pattern in non-mammal herbivores, we conducted feeding trails with 24 tortoises of various species (M range 0.52–180 kg) fed a diet of grass hay ad libitum and salad. Mean daily dry matter and gross energy intake measured over 30 consecutive days scaled to M^0.75 (*95%CI* 0.64–0.87)^ and M^0.77 (*95%CI* 0.66–0.88)^, respectively. Methane production was measured over two consecutive days in respiration chambers and scaled to M^1.03 (*95%CI* 0.84–1.22)^. When expressed as energy loss per gross energy intake, methane losses scaled to 0.70 (*95%CI* 0.47–1.05) M^0.29 (*95%CI* 0.14–0.45)^. This scaling overlaps in its confidence intervals to that calculated for nonruminant mammals 0.79 (*95%CI* 0.63–0.99) M^0.15 (*95%CI* 0.09–0.20)^, but is lower than that for ruminants. The similarity between nonruminant mammals and tortoises suggest a common evolution of the gut fauna in ectotherms and endotherms, and that the increase in energetic losses due to methane production with increasing body mass is a general allometric principle in herbivores. These findings add evidence to the view that large body size itself does not necessarily convey a digestive advantage.

## Introduction

Among the different advantages commonly linked to an increase in body size [1], a widespread concept is that of an increasing digestive efficiency in larger herbivores. Based on the observation that energetic requirements of animals scale to metabolic body mass (i.e., M^0.75^) but gut capacity scales linearly with body mass (M^1.0^) in mammalian herbivores, Bell [2] and Jarman [3] deducted that at larger M, more gut capacity was available per unit energy requirement/food intake. This so-called ‘Jarman-Bell principle’ [4] was further refined subsequently [5]–[7] and has found widespread application in ecology [8]–[11].

This attractive concept provides an intuitive reason for the observation that larger-bodied herbivores usually ingest food of lower nutritional quality [12], [13]. However, recent findings do not support the notion that digestibility [14], [15] or ingesta retention [16] increase systematically with body mass in mammals, and also not in herbivorous reptiles [17]. Among potential disadvantages, ingesta particle size – one of the factors influencing digestive efficiency – increases with body mass [18], [19], and it has been suggested that energetic losses due to methane production are also higher in larger animals [20].

Methane production has been mainly measured in domestic herbivores to address the issue of feed energy use or, more recently, methane mitigation to reduce greenhouse gas emissions [21]. Studies on methane production of non-domestic species have mainly been to complete national or global methane budgets [22]. In contrast, comparative investigations on methane production with respect to herbivore physiology are rare. Methane production has been demonstrated in faeces of captive specimens of nearly all herbivorous terrestrial herbivores, including reptiles [23], and methanogenes have been demonstrated by fluorescence microscopy in land and marine iguanas [24]. In vivo methane production has not been investigated in reptiles to our knowledge. Recently, Franz et al. [25], [26] presented data collections that suggest that methane production scales linearly with M in ruminant and nonruminant mammalian herbivores. The implication of this finding is that because food intake scales to M^0.75^, energetic losses due to methane increase per unit ingested food with increasing body size. Thus, methane energy losses could become a serious constraint in species with large body size. Similarly, allometric relationships were the basis of the investigation of Smith et al. [27] who found that the body mass distribution in a herbivore fauna will impact this fauna's contribution to the global methane budget. Apparantly, methane production scales differently than metabolic requirements or rates.

In order to test the concept of disproportionately increasing methane losses with increasing herbivore M with an original dataset, we chose herbivores of another clade, tortoises. In tortoises, a large range of *M* is available with minimal differences in digestive anatomy and physiology. Scaling of food intake, gut capacity or digesta retention with M is generally similar in herbivorous reptiles and mammals [19], [28]. The aim of our study was to test whether, in tortoises, voluntary food intake scales to M^0.75^, and methane production scales linearly with M.

## Materials and Methods

This study was performed in accordance with Swiss animal welfare legislation (approved by the Cantonal Veterinary Office Zurich under experimental licence number 192/2006). We performed intake and respiration chamber measurements in 24 individual tortoises of the species *Testudo graeca* (n = 5, 1.16±0.95 kg, range 0.52–2.83 kg), *T. hermanni* (n = 6, 1.28±0.36 kg, range 0.91–1.72 kg), *G. nigra* (n = 2, 5.50±0.28 kg, range 5.30–5.70 kg), *Geochelone sulcata* (n = 8, 27.8±18.0 kg, range 7.2–50.0 kg), *Dipsochelys dussumieri* (n = 3, 141±38 kg, range 104–180 kg). Animals were kept individually for 30 days at 27–30°C for intake measurements after an adaptation period of one week. The diet consisted of grass hay and salad in varying proportions; details on intake and digestibility measurements were described previously [17]. Water was available ad libitum at all times. Feed offered and left over was quantified, and faeces were collected completely. Representative subsamples were used to determine dry matter (DM), crude protein, gross energy (GE) and neutral detergent fibre (NDF) concentrations using standard methods [29]; these data allowed the calculation of the apparent digestibility of DM, GE and NDF [30]. Experimental conditions or sample size did not always allow all analyses to be performed for all individuals (cf. Table 1). The ingested diets contained crude protein at 130±18 g kg DM^−1^ (range 95–170) and NDF at 488±107 g kg DM^−1^ (296–662).

**Table 1:** Allometric scaling relationships for tortoises (T), mammalian nonruminants (NR) and ruminants (R) for daily methane production with body mass (M) according to the equation *y* = *a* M*^b^*.

Herbivore group	*y*	unit	n*	*a*	*95% CI a*	*b*	*95% CI b*	*r^2^*	*p*
T	Methane	L d^−1^	24	0.014	0.009–0.023	1.03	0.84–1.22	0.85	<0.001
NR			41	0.181	0.144–0.227	0.97	0.92–1.02	0.98	<0.001
R			62	0.661	0.420–1.040	0.97	0.88–1.07	0.87	<0.001
T		L (kg DMI)^−1^	22	3.02	2.07–4.40	0.33	0.18–0.47	0.52	<0.001
NR			25	3.34	2.63–4.26	0.16	0.10–0.22	0.59	<0.001
R			45	16.58	12.17–22.60	0.12	0.06–0.18	0.25	<0.001
T		L (kJ GEI)^−1^	21	0.70	0.47–1.05	0.29	0.139–0.446	0.46	0.001
NR			25	0.79	0.63–0.99	0.15	0.093–0.204	0.57	<0.001
R			44	3.53	2.52–4.94	0.13	0.058–0.195	0.25	<0.001
T		L (kJ DEI)^−1^	16	0.91	0.51–1.60	0.32	0.13–0.51	0.45	0.003
NR			31	1.48	1.21–1.81	0.17	0.13–0.21	0.71	<0.001
R			35	7.87	5.13–12.06	0.09	−0.001–0.18	0.11	0.053
T		L (g dNDFI)^−1^	21	10.1	6.6–15.5	0.30	0.13–0.46	0.43	0.001
NR			23	11.1	9.1–13.5	0.17	0.12–0.22	0.70	<0.001
R			17	57.4	26.3–125.2	0.11	−0.05–0.27	0.12	0.170

DM dry matter, GE gross energy, DE digestible energy, dNDF digestible neutral detergent fibre, I intake tortoise data from this study; ruminant data collection from Franz et al. [25], nonruminant data collection from Franz et al. [26].

*sample sizes vary between measurements because for tortoises, not all measurements could be performed due to logistic reasons, and because for mammals, data available from the literature varied between sources.

After 30-day intake measurements, tortoises were transferred to open circuit respiration chambers constructed and operated as described in Soliva and Hess [31] for two consecutive 22.5 h periods (temperature 29±1°C, constant humidity 60%, pressure 987±8 hPa; chambers for M from 0.5–10 kg: volume 0.85 m^3^, air flow 1.09±0.08 m^3^ h^−1^; chambers for M from 20–180 kg: volume 4.55 m^3^, air flow 6.08±2.77 m^3^ h^−1^). Animals were measured individually except for the tortoises <5 kg; after pilot measurements, two groups of five individuals between 0.5–2 kg and one group of three individuals between 2–3 kg were measured together, and results divided by the number of animals. Animals had access to feed and water in the respiration chambers. All gas volumes were corrected for standard conditions (1013 hPa, 0°C, 0% relative humidity). Methane concentrations were measured by Binos 1001 (infra-red; Fisher-Rosemount, Baar-Walterswil, Switzerland). Following various conventions in the scientific literature, daily methane production was not only expressed in absolute terms, but also in relation to DM, GE, digestible energy (DE) and digestible NDF (dNDF) intake. Data were analysed after ln-transformation using regression analysis with PSAW 18.0 (SPSS Inc., Chicago, IL), indicating 95% confidence intervals (*95%CI*) according to y = *a* M*^b^* or ln_y_ = ln*_a_*+*b* ln_M_.

## Results

**Figure 1:**
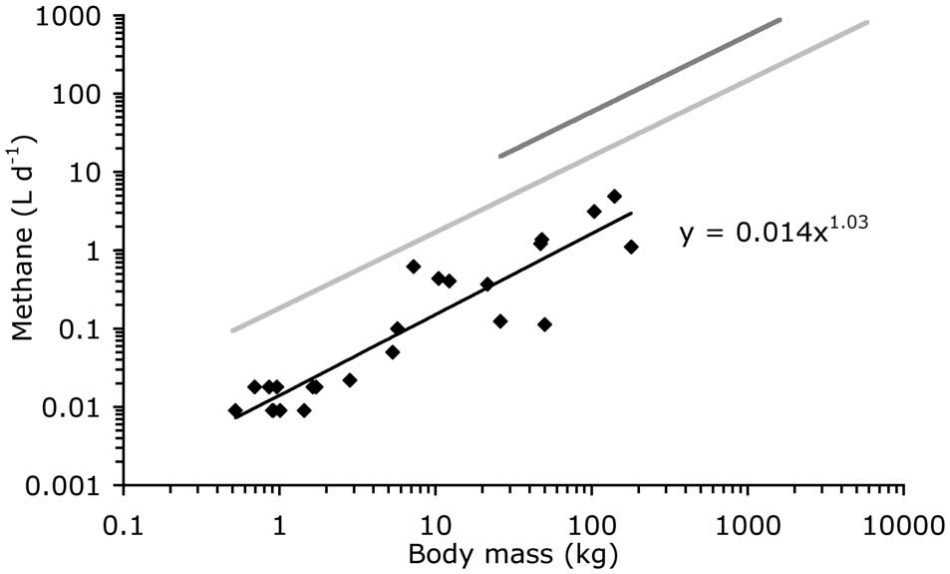
Relationship between body mass and absolute daily methane production; data for ruminants (dark grey regression line; data collection from Franz et al. [**25**]), nonruminant mammalian herbivores (light grey regression line; data collection from Franz et al. [**26**]) and for tortoises in this study.

**Figure 2:**
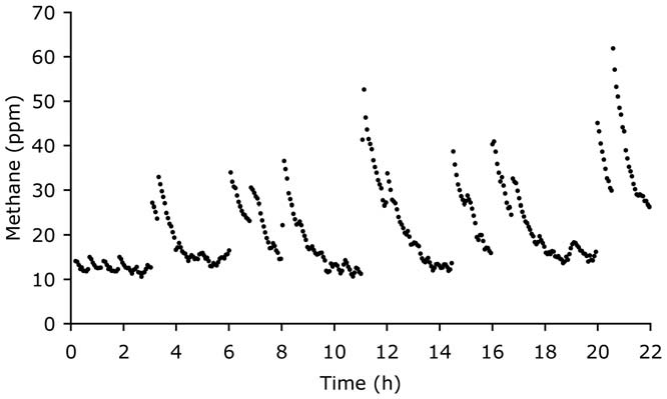
Example of methane production in an open circuit respiration chamber in a *Geochelone sulcata* (10.5 kg) for one uninterrupted measurement period of 22 hours.

Mean dry matter intake (in kg d^−1^) of the tortoises scaled to 0.005 (*95%CI* 0.004–0.007) M^0.75 (*95%CI* 0.64–0.87)^ (*n* = 22, *r^2^* = 0.90, *p*<0.001) and mean daily gross energy intake (in kJ d^−1^) to 86.1 (*95%CI* 64.5–114.7) M^0.77 (*95%CI* 0.66–0.88)^ (*n* = 21, *r^2^* = 0.92, *p*<0.001). In contrast, mean daily methane production scaled linearly to M (Table 1, Fig. 1). During measurements in the respiration chamber, it was noted that methane production was not constant throughout the day but occurred in distinct bursts (Fig. 2).

**Figure 3:**
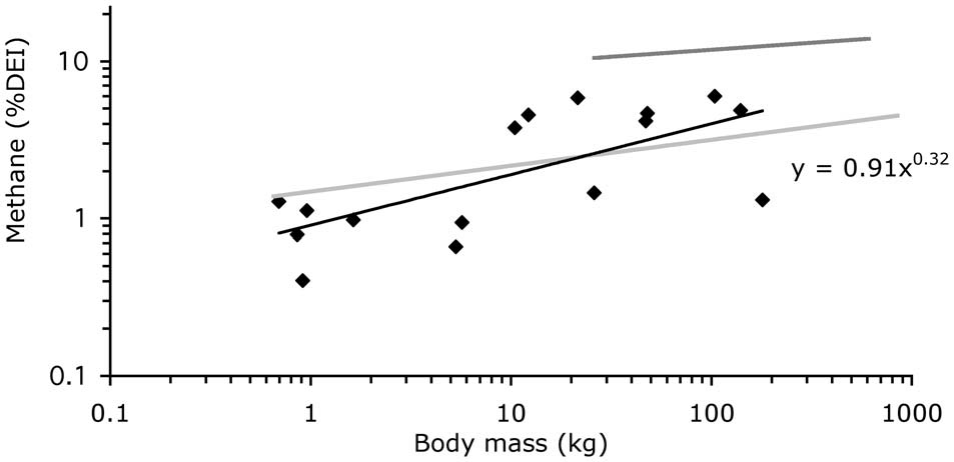
Relationship between body mass and methane energy losses in % of daily digestible energy intake; data for ruminants (dark grey regression line; data collection from Franz et al. [**25**]), nonruminant mammalian herbivores (light grey regression line; data collection from Franz et al. [**26**]) and for tortoises in this study.

**Figure 4:**
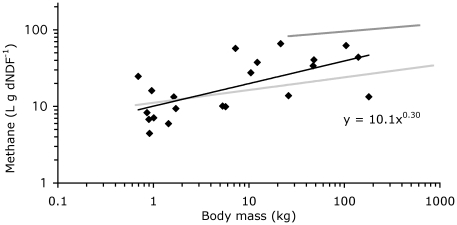
Relationship between body mass and methane energy losses related to the daily intake of digestible cell wall (neutral detergent fibre); data for ruminants (dark grey regression line; data collection from Franz et al. [**25**]), nonruminant mammalian herbivores (light grey regression line; data collection from Franz et al. [**26**]) and for tortoises in this study.

When expressed in relation to intake of digestible energy and fibre, methane losses scaled to M^0.32^ and M^0.30^, respectively (Table 1, Fig. 3 and 4). The *95%CI* of scaling exponent *b* overlapped between tortoises, nonruminant mammals, and ruminants where data had been obtained in previous assessments [25], [26], except for the scaling exponent when methane was related to digestible energy (not significant in ruminants). The *95%CI* of factor *a* was invariably higher in ruminants than in the other two groups (Table 1).

## Discussion

The results of this study suggest that in herbivores, methane production scales linearly with body mass, and the proportional losses of energy from feed ingested due to methane output increase with increasing body mass. Although the existing data must still be considered scarce, the parallel findings in ruminant and nonruminant mammalian herbivores and herbivorous tortoises strongly suggest a general scaling pattern.

Similar scaling patterns in reptiles and mammals have been found for other parameters such as field metabolic rate [32], [33], feed intake [16], [17], [34], or ingesta particle size [19] – although on different levels; whilst some other measures appear relatively similar between herbivorous reptiles and mammals, such as the proportion of the gut contents of total body mass [17], [28] or the achieved digestibilities [35], [36]. Generally, it is assumed that energy metabolism in reptiles is roughly a tenth of that observed in mammals [37]. The difference in the intercept *a* of the regression equation describing dry matter intake in the tortoises of this study (0.005) compared to the intercept of 0.047 found in herbivorous mammals in general [16] fits this pattern, as does the difference in the intercept describing the absolute methane output (0.014 in tortoises vs. 0.181 in nonruminant mammals, Table 1). Consequently, when methane production is expressed per unit intake, there is no significant difference in the intercept *a* between tortoises and nonruminant mammals (Table 1).

This finding indicates a common adaptation of the gastrointestinal fauna between ectotherms and endotherms. Other similarities between the microbial faunas of herbivorous reptiles and mammals have been reported, such as the number of gut bacteria and the presence of protozoa [38]–[40], cellulase activity [41], or the concentration of fermentation products [42]–[45]. A relatively similar methane production per unit food intake in reptiles and mammals means that the processes of microbial fermentation must be similar even though the microbial faunas of reptiles and mammals will vary distinctively in their temperature sensitivity. The findings suggest that methane production is a more or less constant, unavoidable by-product of microbial fermentation in herbivores. Because of the well-documented differences in ingesta retention times between herbivorous reptiles (230±140 h [17], [46]) and mammals (40±25 h [16]), the similarity in methane scaling between reptiles and mammals also indicates that retention time as such is not the main factor influencing the scope of methane production, even if it may be relevant when comparing data within species [47], [48]. Our results also suggest that the increase in methane production with increasing body size is not only due to an increase in fibre digestibility at higher body sizes; when expressed per unit of digestible fibre intake, the effect of an increasing methane production remains and scales similarly with M as when expressed in relation to other intake measures (Table 1).

Prins and Kreulen [49] and Van Soest [50] suggested that a different group of methanogenes – slower-growing archeae with a generation time of about 4 days that produce methane from acetate in sewers, for example – may actually limit body size in herbivores. They considered ingesta retention a function of body mass [6], [7], [16] and hypothesized that when retention times surpass 4 days, energetic losses due to acetate-based methanogenesis would become prohibitve for the host. In herbivorous reptiles retention times beyond 96 h are common [46], [51] which indicates that other factors than retention time must limit the occurence of slow-growing archeae in herbivores.

An interesting question is could methane production by the fast-growing archeae be a constraint on the evolution of body size? This has been suggested for ruminants, due to the high proportion of energetic methane losses in this group [25]; for nonruminant mammals, these losses might become limiting at extrapolated body masses of 100 metric tonnes [26] – a putative constraint that might apply conceptually for the largest dinosaurs [1]. Reptiles never reached such proportions. When the regression equation from tortoises is directly applied to the largest known chelonian, *Archelon ischyros*, a marine turtle with an estimated maximum M of 5000 kg [52], extrapolated methane energy losses per unit of digestible energy intake (14%) approach those found in large ruminants. Note that this similarity to ruminants, in spite of the general similarity in scaling between tortoises and nonruminant mammals, is due to the determined exponent *b* of 0.32, which is numerically higher than the one calculated for nonruminant mammals (0.17), though overlapping in its confidence interval. Differences in exponent should be considered with caution when extrapolations beyond the M range are performed that served to generate the regression equation [28].

Why herbivores apparently did not evolve to avoid methane losses is a fundamental question. Intervention studies in domestic ruminants have shown that functional digestion can be maintained in the absence or near-absence of Archeae and without methane production [53]–[56]. An alternative view of methanogenes could be that they are among the prerequisites for herbivory. Pimentel et al. [57] showed that, in a models with dogs and guinea pigs, methane slowed intestinal passage by decreasing intestinal contractile activity. In humans, methane production is associated with increased digesta retention times [58]–[61], and is positively correlated with constipation and negatively with diarrhoea [62], [63]. Reduction of methane production by oral antibiotic treatment leads to a reduction of constipation [64], [65]. While offering new insights into potential therapeutical interventions against human irritable bowel syndrome, these results also give rise to the speculation that the presence of methane, and its passage-delaying effect, was an important component of the evolution of physiological adaptations to herbivory, which requires long passage times. However, confirmation of this hypothesis requires much further research.

Our study shows that methane losses not only occur in mammalian but also in reptilian herbivores, and that they scale linearly with body mass, thus representing proportionally increasing losses at increasing body size. Therefore, differences in the proportion of ingested energy lost to methane, according to the body size composition of any mammal or reptile herbivore fauna should be considered when reconstructing trophic energy fluxes in ecosystems, or contributions of these ecosystems to changes in the composition of the atmosphere [27]. Further studies combining in vivo measurements and microbiological analyses should unravel the fundamental principles behind the link between microbial fibre fermentation in vertebrate herbivores and methane production.
